# Fishing-induced life-history changes degrade and destabilize harvested ecosystems

**DOI:** 10.1038/srep22245

**Published:** 2016-02-26

**Authors:** Anna Kuparinen, Alice Boit, Fernanda S. Valdovinos, Hélène Lassaux, Neo D. Martinez

**Affiliations:** 1 Department of Environmental Sciences, P.O. Box 65, 00014 University of Helsinki, Finland; 2 Potsdam Institute for Climate Impact Research, P.O. Box 60 12 03, 14412 Potsdam, Germany; 3 Department of Ecology & Evolutionary Biology, P.O. Box 210088, The University of Arizona, Tucson, AZ 85721, USA; 4 Pacific Ecoinformatics and Computational Ecology Lab, 1604 McGee Avenue, Berkeley, CA 94703, USA; 5 École Polytechnique, Route de Saclay, 91128 Palaiseau, France

## Abstract

Fishing is widely known to magnify fluctuations in targeted populations. These fluctuations are correlated with population shifts towards young, small, and more quickly maturing individuals. However, the existence and nature of the mechanistic basis for these correlations and their potential ecosystem impacts remain highly uncertain. Here, we elucidate this basis and associated impacts by showing how fishing can increase fluctuations in fishes and their ecosystem, particularly when coupled with decreasing body sizes and advancing maturation characteristic of the life-history changes induced by fishing. More specifically, using an empirically parameterized network model of a well-studied lake ecosystem, we show how fishing may both increase fluctuations in fish abundances and also, when accompanied by decreasing body size of adults, further decrease fish abundance and increase temporal variability of fishes’ food resources and their ecosystem. In contrast, advanced maturation has relatively little effect except to increase variability in juvenile populations. Our findings illustrate how different mechanisms underlying life-history changes that may arise as evolutionary responses to intensive, size-selective fishing can rapidly and continuously destabilize and degrade ecosystems even after fishing has ceased. This research helps better predict how life-history changes may reduce fishes’ resilience to fishing and ecosystems’ resistance to environmental variations.

Commercial fishing is widely thought to magnify fluctuations in the abundance of the targeted population as suggested by variations in larval abundances^1^. While such fluctuations could, in principle, arise from varying fishing pressure or increased environmental variation, these fluctuations have been found most clearly correlated with shifts of fish life histories towards young, small, and more quickly maturing individuals^2^. Namely, age truncation of populations can lead to increasing instability in population dynamics owing the changes in intrinsic population growth rates^2^. However, the existence and nature of the mechanistic basis for the correlation between population age and size structure and instability of population dynamics as well as its potential ecosystem impacts remain poorly understood.

Fishing alters age and size composition of the targeted population directly through removal of those ages and sizes specifically selected by fishing gears. However, numerous commercially exploited fish populations show not only demographic shifts in population structure but also trends in fish life-histories towards earlier maturation and declining adult body size^3^. While such changes can be also induced by increasing water temperatures^4^, most of the observed trends correlate positively with fishing pressure suggesting that the phenotypic changes are induced by fishing and potentially be, or at least resemble, evolutionary responses to fisheries-induced selection^5^,^6^. Quantifying ecological feedbacks of such life-history changes are key to understanding how these changes translate into species’ viability and recovery^7^. Such understanding may help people to manage the stability of fish populations including their resistance and resilience to exploitation and natural disturbances.

Here, we use a network approach^8^ to mechanistically explore the effects of fisheries-induced shifts in fish demography and life history on the dynamics of harvested species and the ecosystem. We elucidate potential causes of such effects by comparing ecosystem dynamics across periods before, during, and after fishing, in the absence and presence of life-history changes induced by fishing. We further explore the responsible mechanisms by disentangling the relative roles of life-history changes in fishes’ body size and maturation as well as the demographic effects of fishing mediated through biomass removal and fishing selectivity. The robustness of the results derived based on deterministic food web dynamics is also explored with respect to alternative levels of environmental stochasticity associated with ecosystem primary productivity. Our methods integrate two different and well-established components that model basic mechanisms responsible for the abundance of fishes in ecosystems. One component describes food-web dynamics^9^. The other describes how fishing impacts vary with the life stage of the fishes. Food web dynamics are based on consumer-resource interactions where the abundance of a species is determined by the balance of gains from consuming resources (e.g., feeding) and losses to metabolism and other consumers (e.g., predators^10^,^11^). Key parameters such as metabolic and feeding rates are widely measured and remarkably well estimated from each species’ mean body size^12^,^13^.

An allometric trophic network model^14^ (ATN) based on this approach has been extended and specifically parameterized for the Lake Constance (LC) ecosystem at the Northern foot of the European Alps^9^. The LC ATN model accurately simulates the seasonal dynamics of the plankton community’s biomass and productivity including 20 functional guilds of bacteria, phytoplankton, and zooplankton fed upon by 4 functional guilds of fish^9^. Here, we extend the LC ATN model by separating each of the two most common and commercially fished species in LC-the piscivorous Eurasian perch (*Perca fluviatilis*; hereafter denoted as perch) and planktivorous whitefish (*Coregonus lavaretus*)-into five life stages (total of 10 functional guilds) including larvae, juveniles, 2 years, 3 years, and 4 years or older (4+) (Fig. 1). We model the dynamics of these stages by modifying a size-structured biomass model that is a less fully age-structured version of a physiologically-structured population model^15^. In contrast to the original version’s application of all adults’ energy beyond maintenance costs to reproduction, our modification allows adults to continue growing while also reproducing. We parameterized the stages using empirically measured growth parameters of the species in LC and a widely used and empirically accurate model of body size as a function of the fishes’ age^16^. Fishing-induced life-history changes were estimated using empirical observations of the effects of fishing on body size and maturation age^6^,^17^. Fishing mortality was parameterized according to observed fishing pressure in LC^18^.

## Results

We allowed our *in silico* ecosystem to reach a dynamic equilibrium and then exposed it to fishing for 50 years, after which the populations were allowed to recover in the absence of fishing. Compared to unexploited abundances, fishing decreased mean biomasses of whitefish by 10–20% and perch by about 40% (Fig. 2). The deterministic ecosystem dynamics express intrinsic periodic fluctuations whose magnitudes are affected by fishing and fish life-history changes. Fish species exhibited higher fluctuations in total population biomasses under fishing as compared to the pre-fishing period (for whitefish, standard deviations (SD) and coefficients of variation (CV) were 20–50% and 50–70% higher, respectively; for perch SDs and CVs were 95–100 times larger during fishing). For both species, variability was similar in the presence and absence of life-history changes. In whitefish, life-history changes caused a further decline in mean biomass by about 10% as compared to the fishing scenario without life-history changes. The corresponding decline owing to life-history changes in perch was relatively minor. However, after fishing had ceased, the populations with life-history changes recovered to mean biomasses summed over all of each species’ life stages that were 12% (whitefish) and 4% (perch) lower than in the populations lacking life-history changes.

As one may expect, the largest biomass losses (~70% decline) during fishing were in the oldest (4+) age class that is most heavily fished among both whitefish and perch. Younger age classes were less affected and 2-year-old whitefish even increased their abundance by ~30% during fishing. In the absence of life-history changes, all stages recovered to their earlier unexploited densities after the fishing was relaxed. However, in the presence of life-history changes, the biomasses of whitefish and perch in the oldest age class recovered to abundances that were 21% and 8% lower, respectively, than their original unexploited biomasses. Effects of life history changes on the recovered biomasses of 2 and 3 year old fish were minor (Supplementary Fig. S1 online).

The LC whitefish population is sustained by the hundreds of thousands of offspring of LC adults being reared in a hatchery and released into LC. Our model includes this large input and predicts that the energy whitefish invest into natural reproduction is approximately 100 times less than the hatchery input. Given this intervention, we focus further consideration on the less artificially supplemented perch population when exploring the role of life-history changes on juvenile production and on the resilience of populations to fishing.

Energy that perch invested annually into juvenile production increased during fishing as inter- and intraspecific competition relaxed whether or not life history changes accompanied the fishing. The presence of life history changes further increases energy investment in juveniles by ~100% during the last 10 years of fishing and this investment remains about ten times higher as the population recovers in the absence of fishing. This increased investment results from the maturation of younger, partially planktivorous perch, such that a wider range of perch age classes which consume a broader range of prey contribute to juvenile production. An even more striking consequence of the life-history changes than the increase of investment into juveniles was the large increase in investment variability (Fig. 2). SDs of the year-to-year variability of energy invested into juvenile production were 60% larger than those in the absence of life-history changes (whereas CV was about 20% lower owing to heavy increase in average investment). Increased fluctuations in perch reproduction reflect variability in the amount of ingested energy allocated for juvenile production which is associated with increased variability of the plankton groups consumed by 2 and 3 year old perch (Fig. 3).

Fishing induced life-history changes also dramatically increased the variability of the total ecosystem biomass both in the presence and absence of fishing (Fig. 2). Compared to the absence of life-history changes, the presence of such changes caused the SD and CV of the total ecosystem biomass in the middle of the growth season to be 72% and 75% larger in the last ten years of fishing and 140% and 157% larger in the recovered populations, respectively. Life-history changes also decreased the recovered ecosystem biomass by ~6%. If viewed in the end of each growing season, life-history changes increased SD and CV of total ecosystem biomass by ~100% in the last ten years of fishing and by ~30% in the recovered population, but did not affect the recovered average biomass. Variability seen at the ecosystem level mirrors the increased variability in five out of six major plankton groups in the LC ecosystem. Fish life-history changes increased year-to-year fluctuations in the five plankton components even after fishing ceased and fish populations had recovered most of their pre-fished biomasses (Fig. 3). Almost all of these broad changes in variability are due to decreased body sizes whereas maturation had relatively little effect (Fig. 2). This conclusion is further supported by the observation that if 2, 3, and 4+ year old fish were harvested at the same rate (rather than selectively targeting older fish; see methods), variability increases associated with fishing were smaller than when larger sizes were selected for (Supplementary Fig. S2, S3 online). This shows that biomass removal by fishing alone can increase variability of fishes, but the demographic effect due to preferential removal of larger, older individuals by fishing can degrade and destabilize the harvested ecosystem by further shifting population’s body size distributions towards smaller individuals. These impacts are magnified and extended beyond immediate exposure to fishing by fisheries-induced life-history changes (i.e. reducing age-specific body size and maturation age), which decrease fishes’ body sizes even more.

Adding moderate environmental stochasticity by adding a SD = 10–15% to the phytoplankton’s carrying capacity (*K*) did not completely mask the overall effects of life-history changes on fishes, their reproduction, and the total ecosystem biomass. However, impacts of fish life-history changes on plankton community became less evident and were nearly completely masked by year-to-year variability with the standard deviation 15% of *K* (Supplementary Fig. S4, S5 online). In the absence of biomass removal by fishing, the consequences of fish life-history changes were analogous to those under fishing. This shows that species and ecosystem feedbacks of earlier maturation and declining age-specific fish body sizes do not depend on density-dependent processes mediated by the removal of biomass due to fishing (Supplementary Fig. S6, S7 online).

Results derived using alternative fishing pressures and rates of evolution (see methods) gave analogous results to those presented here (results not shown). Higher and lower rates of evolution lead, respectively, to larger and smaller amounts of variability in reproduction and in the ecosystem as well as in larger and smaller reductions in the abundance of 4+ age class in the recovered populations. Higher and lower fishing levels slightly increased and decreased, respectively, fishing-induced reductions in 4+ age class abundance and had much less impacts on other age classes. Changing fishing pressures and evolution rates did not alter the relative roles of body-size and maturation components of life history changes.

## Discussion

The present study demonstrates how fluctuations in fished populations may be magnified by declining fish body size coupled with advanced maturation and how fluctuations may extend well beyond targeted species to the whole ecosystem long after fishing has ceased. Moreover, life-history changes appear to erode fish biomass by substantially decreasing the proportion of large, old individuals. Given their vital role in producing good quality juveniles^19^ and in stabilizing populations^2^, life-history changes are likely to reduce population viability and its ability to buffer against and recover from disturbances and environmental fluctuations^20^,^21^.

While previous studies have identified destabilizing effects of fishing^1^ and associated destabilization with a list of various changes in life histories of fish populations^2^, we go further here by demonstrating and quantifying how different changes within such lists may alter the intrinsic dynamics of consumer-resource networks^11^,^12^,^13^,^14^ to generate empirically observed increases in variability. Contrary to the previous analyses asserting that life-history changes alone are not likely to generate instability in the absence of environmental variations^22^, we demonstrate that the feedbacks of these changes mediated by species interactions appear sufficient to increase fluctuations in the deterministic dynamics of the harvested species and the ecosystem. Simply removing large, old individuals by fishing increases fluctuations in the target populations’ dynamics and in the entire ecosystem. When direct demographic effects of fishing are coupled with changes in fish life-histories towards earlier maturation and smaller age-specific body sizes, the fluctuations are substantially magnified and change the dynamics of the harvested ecosystem even long after fishing has ceased. Our results suggest that effects of maturation mechanisms primarily involve increases in larval variability while nearly all other ecosystem-level and long-term feedbacks are largely attributable to mechanisms associated with body size. This outcome is more generally predicted by theory indicating that decreased body-size ratios of consumers and resources destabilizes populations and ecosystems^12^,^13^. The applicability of this prediction to the specific parameter spaces of ecosystems exposed to fishing had not previously been demonstrated.

Real ecosystem dynamics are clearly also affected by numerous external environmental drivers not accounted for in our deterministic models where all variability is driven by intrinsic dynamics of the system. Nonetheless, our findings still provide important insights into the mechanisms and pathways through which fishing and associated life-history changes can affect the harvested ecosystem. The signal of such impacts in empirical data can be masked by sampling errors and stochasticity arising from the environmental variations. Given such challenges, mechanistic investigations such as ours provide guidance for understanding the dynamics and detecting cryptic signals within harvested ecosystems. We show that our findings mirror previous empirical observations^2^ even in the presence of moderate environmental stochastic in plankton growth. However, since environmental drivers and noise can alter and interact with mechanistic dynamics of ecosystems in many other ways not addressed here, our findings do not eliminate other potential causes of the increased fluctuations seen in fished populations. Instead, we more humbly suggest how specific life-history changes may currently be the most plausible and empirically well-supported mechanism responsible for such increases.

More broadly, our study touches upon ‘cohort resonance’ in age-structured populations^23^, which may be responsible for increased variability due to fishing^24^. Truncating the ages of spawning individuals can exacerbate variability in fish populations due to cohort resonance^24^. Our study is consistent with this observation and goes further by disentangling responsible mechanisms: increased overall mortality, selective removal of large, old individuals, and changes in fish life-histories are all capable of increasing larvae fluctuations. While in real fisheries these pathways would occur in parallel, each of these three components alone appears sufficient to add fluctuations. Importantly, these effects are mediated and mirrored by the ecosystem, underscoring the importance of such multispecies modelling approaches that are capable of generating fluctuating dynamics, in order to understand and account for fluctuating behaviour of fish stocks subject to fishing^24^.

The needs to manage fisheries in an ecosystem-based manner^25^ and to account for life-history changes associated to fishing are widely acknowledged^26^,^27^. Our work shows how general ecological theory successfully applied elsewhere^9^,^12^,^14^ can also help to achieve these management goals. More specifically, our application of food web models parameterized by well-measured estimates of ecosystem dynamics helps to illuminate mechanistic underpinnings, management options, and dynamic consequences of industrial scale fishing. In particular, our results demonstrate the widely accepted assumption that mechanisms responsible for variability of juveniles are the same as those responsible for adult variabilty^1^,^2^ may not be valid. In contrast, our results suggest that managing for later maturing adults will reduce juvenile variability and managing for larger sized adults will create more abundant, stabile and resilient adult fish populations. More generally, we show how dynamics during fishing and recovery emerge from an intriguing combination of feeding interactions and life-history feedbacks, which substantially affect numerous species indirectly. The effects of these dynamics on ecosystems and their services suggest that further exploration of such dynamics as well as their external drivers may substantially help to manage the sustainability and stability of ecosystems on which we critically depend.

## Methods

### Allometric trophic network model (ATN)

**Figure 1:**
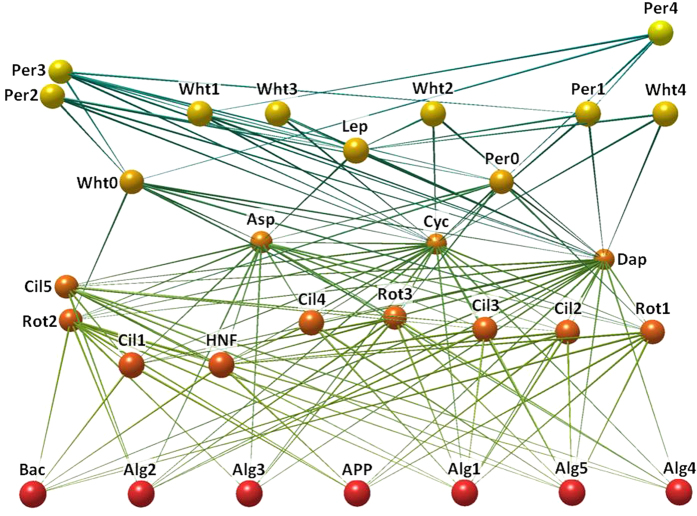
Lake Constance food web. Five guilds of algae (Alg1-5), bacteria (Bac), autotrophic picoplankton (APP), heterotrophic nanoflagellates (HNF), five guilds of ciliates (Cil1-5), four guilds of rotifers (Rot1-3, Asp), herbivorous crustacean (Dap), carnivorous crustacean (Cyc, Lep), whitefish larvae (Wht0), perch larvae (Per0), whitefish juveniles (Wht1), perch juveniles (Per1), and 2, 3, and 4+ year old whitefish (Wht2-Wht4) and perch (Per2-Per4). Labels of each node correspond to the guild ID in Supplementary Table S1 online. Colours change from red to yellow along with increasing trophic level of the guild. Drawn using Network3D software^35^,^36^.

**Figure 2:**
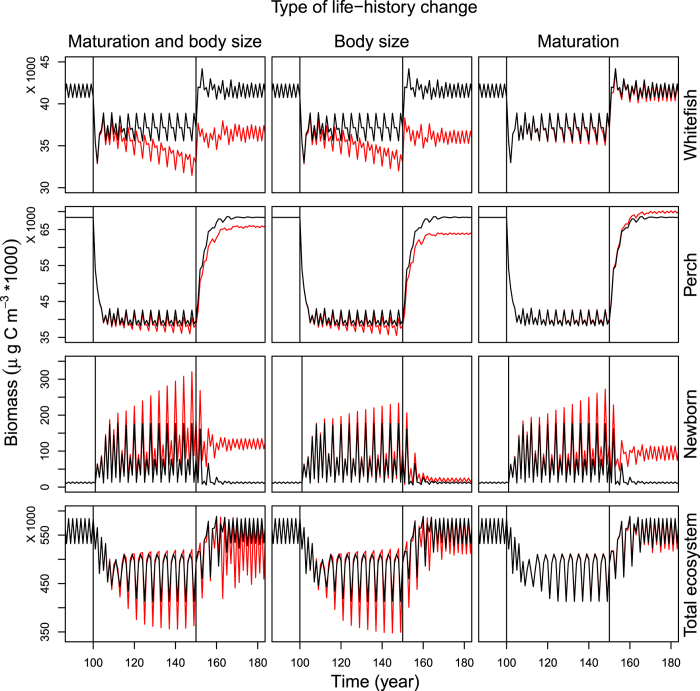
Simulated time series for fish and ecosystem biomasses. Annual biomasses of whitefish, perch, and newborn perch at the end of each growing season, as well as the total ecosystem biomass in the middle of the growth season are shown by solid lines. Scenarios for the presence and absence of life-history changes are indicated with red and black, respectively. The beginning and the end of fishing are indicated by vertical lines. The first panel column shows the combined effect of the reduction in fishes’ age-specific body sizes and the age at maturation during fishing, while the second and the third column separate these effects, such that either age-specific body sizes reduce during fishing in the absence of changes in maturation (the second column) or the age at maturation reduces during fishing in the absence of changes in fishes’ age-specific body sizes (the third column).

**Figure 3:**
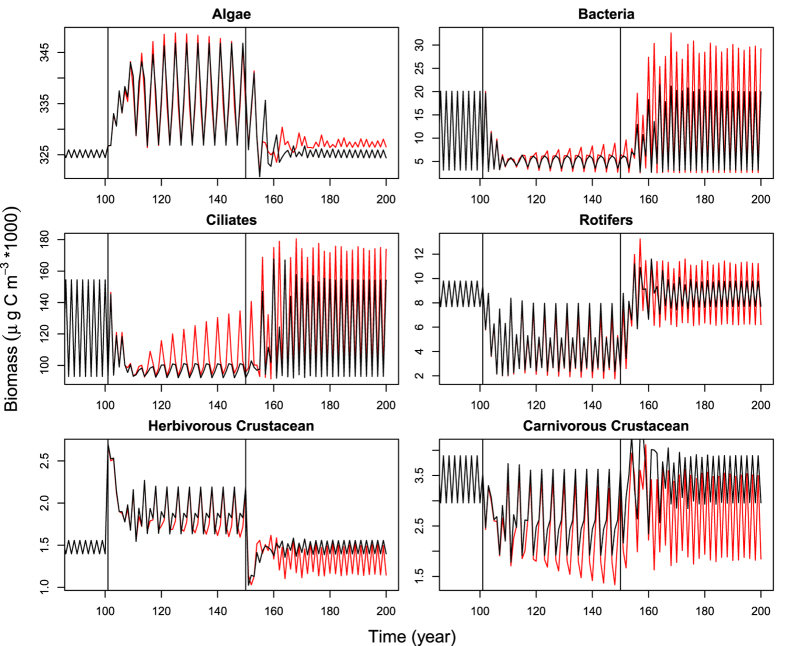
Simulated time series for six groups of plankton at the end of each growing season. Scenarios for the presence and absence of fish life-history changes are indicated with red and black, respectively. The beginning and the end of fishing are indicated by vertical lines. See legend of Fig. 1 for the aggregation of the guilds into the plankton groups.

We use the M2 version of the ATN model in ref. 9 without abiotic forcing. This ATN model is based on a set of ordinary differential equations (ODE) originally developed and applied to a 3-species food chain^10^, and later extended to *n*-species^11^,^28^,^29^ and then further extended to include detritus and growth inefficiencies^9^. The following slightly modified equations of ref. 9 for (1) producers, (2) consumers, and (3) detritus form the basis of the extension we develop here:


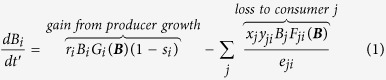



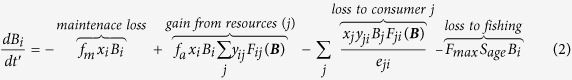



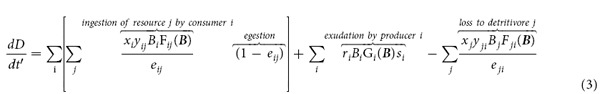


where ***B*** refers to the matrix of all biomasses, *B*_*i*_ is the biomass of guild *i*; *r*_*i*_ is intrinsic growth rate of producer *i*, *G*_*i*_*(**B**)* is logistic growth 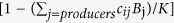 including producer competition coefficients 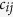 and carrying capacity *K* shared by all autotrophs; *s*_*i*_ is the fraction of exudation; *x*_*i*_ is the mass-specific metabol*i*c rate of consumer *i* determined based on allometric scaling; *y*_*ij*_ is the maximum consumption rate of guild *i* feeding on *j*; and *e*_*ji*_ is the assimilation efficiency describing the fraction of ingested biomass that is actually assimilated; *f*_*m*_ is the fraction of assimilated carbon respired by maintenance of basic bodily functions; and *f*_*a*_ is the fraction of assimilated carbon used for production of consumers’ biomass (1- *f*_*a*_ is respired). *F*_*ij*_ (***B***) in Eqn. 3 is the consumers’ normalized functional response


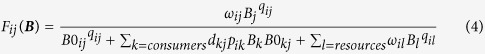


where *ω*_*ij*_ is the relative prey preference of consumer species *i* feeding on resource species *j*; *q*_*ij*_ = 1.2 which forms a relatively stable functional response intermediate between the Holling Type-II and Type-III functional responses^11^; *B0*_ij_ is the half saturation constant of resource species *j* at which consumer species *i* achieves half its maximum feeding rate on species *j*; *d*_*kj*_ is the coefficient of feeding interference of species *k* with *i* while feeding on species *j*; *p*_*ik*_ = the fraction of resource species shared between species *i* and *k. d*_*kj*_ also accounts for prey resistance to consumption that may increase with increasing abundance of consumers of species *j*. See Fig. 1 and Supplementary Methods online, and Supplementary Tables SI and S2 online for further details and a summary of the key model and plankton parameters.

We extended the simple fishing mortality of ref. 9 to include the fishing mortality of the fully selected individuals (*F*_*max*_) and age-specific fishing selectivity (*S*_*age*_). For fish juveniles (*age* = 1) and larvae (*age* = 0) as well as all the organisms that are not fished, *S*_*age*_ = 0. For fish 2 years or older (*age* > 1), selectivity varies logistically according to *S*_*age*_ = 1/ [1 + e^*−2(age-ageF50)*^] (S_age_ is 0.12, 0.50, and 0.88 for age-classes 2 year, 3 years, and 4 year and older, respectively), where *ageF50* is the age at which the relative fishing selectivity is 50% and was set to 3 year for both fish species. This selectivity scenario was chosen to mimic the standard attempt of fisheries management (and gear regulations) to set targets for fishing pressure so that fish may adequately reproduce prior to being caught.

### Fish life-histories and population dynamics

Fish life stages were constructed based on the widely used von Bertalanffy growth model


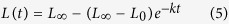


where *L*(t) is the length at age t, *L*_*∞*_ is the asymptotic body length, *L*_*0*_ is the length at t = 0, and *k* (year^−1^) is the intrinsic growth rate at which *L*_*∞*_ is reached^16^. The following growth parameter values were obtained from FishBase (fishbase.org; Accessed: 17th Nov 2015) for whitefish and perch, respectively: *L*_*∞*_ = 52.8 cm and 29.3 cm, *L*_*0*_ = 1.2 cm and 0.7 cm, and *k* = 0.27 year^−1^ and 0.35 year^−1^. Estimates for whitefish are specific to LC whereas perch parameters were obtained from a study that reported averages across 25 lakes in Germany. Length-weight (cm-g) relationships *W* = 0.005 × *L*^3.14^ for whitefish and *W* = 0.0105 × *L*^3.11^ for perch were also obtained from FishBase. To estimate metabolic rates for each life stage, weights (g) were first converted into μgC by multiplying *W*_*c*_ = 0.20 × 0.53 × 10^6^ *W,* assuming that fish dry weight is 20% of its fresh weight^30^ and that 53% of fish dry weight is carbon^31^. The metabolic rate *x* (day^−1^) was then assigned as *x* = 0.88 × (6.4 × 10^−5^/*W*_*c*_)^0.11^, where 0.88 is the metabolic scaling constant for ectotherms^11^, 0.11 is the allometric scaling exponent for fishes^32^, and 6.4 × 10^−5^ corresponds to the body size (g) of the reference guild, whose growth rate is scaled to 1 day^−1^ (SI). Because of the smaller body sizes, perch metabolic rates (0.05–0.16 day^−1^) were slightly higher in each life stage as compared to whitefish (0.04–0.14 day^−1^). The lengths, weights, metabolic rates, and diets for whitefish and perch life stages are given in Supplementary Table S3 online.

The fraction of fish that were mature at each life stage (for ages 2 year, 3 year, and 4+year) was determined based on a logistic equation 1/(1 + e-3*^(age-*ageM50)*^), where *ageM50* is the age at which 50% of fish are mature and was initially set to 3 years for both the species. The age-specific fractions mature were 4.7%, 50.0%, and 95.2% for 2 year, 3 year, and 4+ year old fish, respectively. Larvae and juveniles were always considered immature. The formulation was chosen to mimic the fact that, in LC, most 2 year perch and whitefish are immature, about 50% of 3 year old fish are mature, and most 4+ old fish are mature. In contrast to the plankton community dynamics, fish were assumed to reproduce as a pulse in the beginning of each growth season. While immature fish invest all their surplus energy (assimilated carbon minus respiration) to somatic growth, mature fish were assumed to allocate their energetic investment between growth and juvenile production. More specifically, mature fish aged 2, 3, and 4+ years respectively invested 10%, 15%, and 20% of surplus energy to reproduction and the rest to growth such that fish growth is indeterminate and continues after sexual maturity^33^. Reproductive investment accumulated during each growth season (90 days^9^) becomes larvae biomass in the beginning of the next growth season. Reproductive output equals zero when species gain no surplus energy. Perch and whitefish populations in LC are greatly subsidized by hatchery raised juveniles on whom whitefish are particularly dependent. We addressed this by adding newborn juvenile biomass equivalent to the estimated annual hatchery input to both larvae fish populations (300 μgC/m^3^ for whitefish, 50 μgC/m^3^ for perch; R. Eckmann, *personal comm.*) each spring. Transfer of biomass from each life stage to the next occurred in the beginning of each growth season.

### Simulation design

We simulated the effects of fisheries-induced changes in life histories (advanced maturation, decrease in age-specific body sizes) to investigate their ecological feedbacks on fish population dynamics and the ecosystem. Towards this end, we first allowed the ecosystem to settle into a dynamic equilibrium (100 years) and then simulated a period of fishing (50 years) followed by a period of recovery in the absence of fishing (50 years). Fishing pressure on LC whitefish is estimated to be approximately *F* = 0.5 (year^−1^)^18^, so we focussed on instantaneous fishing mortality rate *F*_*max*_ = 0.5 (year^−1^) in the fishing scenario as being empirically based, but checked the results robustness with scenarios *F*_*max*_ = 0.4 (year^−1^) and *F*_*max*_ = 0.6 (year^−1^) as alternatives. During the period of fishing, we reduced the fishes’ age at 50% maturity (*ageM50*) by 0.5% per year and increased the fishes’ metabolic rate (*x*_*i*_) by 0.0001 per year. The latter corresponds to 0.5% per year decrease in the length-at-age. The rate of 0.5% for life-history changes is relatively conservative and falls well within the range of empirically observed rates of life-history changes in commercially exploited fish populations at *F*_*max*_ = 0.5^5^,^6^. The chosen rate also mimics the reported whitefish body size decline rate in LC over the past decades^17^. In the absence of similar knowledge about perch fishing pressure and potential life-history changes, we applied the above parameterization for both the fish species. Given that evolutionary recovery from fishing is expected to be very slow^34^, we kept life-histories unchanged during the period of recovery. Simulations with the changes in *age*_*50*_ and metabolic rate were compared to similar simulations where maturation and metabolic rate did not change during fishing. Robustness of the results was checked by considering two alternative rates of life-history change, 0.25% and 0.75% per year. To disentangle the relative roles of changes in maturation and body size, simulations were repeated with either age-specific body sizes or maturation changing and compared against the original scenarios. The impacts of mortality and fishing selectivity were disentangled by comparing selective fishing to a scenario with similar total biomass removal, but with fishing pressure distributed equally among 2, 3 and 4+ year old fishes. We compared density-dependent effects of fishing to those mediated by life-history changes by changing maturation and body size from year 100 to year 150 as occurred during the fishing scenario, but with fishing pressure set to zero. Finally, we explored how easily the impacts of life-history changes were masked by environmental stochasticity, by adding noise to the annual phytoplankton carrying capacity (*K*). The noise term was drawn from a normal distribution with the mean zero and three alternative scenarios for the standard deviations (5%, 10%, and 15% of *K*).

## Additional Information

**How to cite this article**: Kuparinen, A. *et al.* Fishing-induced life-history changes degrade and destabilize harvested ecosystems. *Sci. Rep.* **6**, 22245; doi: 10.1038/srep22245 (2016).

## Supplementary Material

Supplementary Information
