# Corrigendum: Fishing-induced life-history changes degrade and destabilize harvested ecosystems

**DOI:** 10.1038/srep41466

**Published:** 2017-02-06

**Authors:** Anna Kuparinen, Alice Boit, Fernanda S. Valdovinos, Hélène Lassaux, Neo D. Martinez

Scientific Reports 6: Article number: 2224510.1038/srep22245; published online: 02 26 2016; updated: 02 06 2017

In the ‘Fish life-histories and population dynamics’ section of this Article the authors erroneously cited a personal communication with R. Eckmann. Dr. Eckmann did not provide any information about perch hatchery and the applied values for whitefish hatchery were modified from raw hatchery larvae records provided by him. The authors apologise for this misinterpretation. Thus, in the simulations the perch hatchery scenario was not based on empirical data but the scenario was constructed to resemble a low level of perch hatchery input as compared to whitefish hatchery input. Nonetheless, the results of the present study are robust to the assumption that perch was or was not subsidized by hatchery input as well as to variations in whitefish hatchery input. Feeding interactions among whitefish and perch are based on knowledge of the species’ ecology in typical Northern hemisphere lakes where the species co-exist, but not from direct fish diet observations from Lake Constance ecosystem.

