# Supplementary material for: Fishing-induced life-history changes degrade and destabilize harvested ecosystems
Source: Sci Rep. 2016 Feb 26;6:22245. doi: 10.1038/srep22245 (PMC4768105; doi:10.1038/srep22245)

Supplementary Information

Fishing-induced life-history changes degrade and destabilize harvested ecosystems

Anna Kuparinen[1*], Alice Boit[2], Fernanda S. Valdovinos[3,4], Hélène Lassaux[5], Neo D. Martinez[3,4]

[1] Department of Environmental Sciences, P.O. Box 65, 00014 University of Helsinki, Finland
[2] Potsdam Institute for Climate Impact Research, P.O. Box 60 12 03, 14412 Potsdam, Germany
[3] Department of Ecology & Evolutionary Biology, P.O. Box 210088, The University of Arizona, Tucson, AZ 85721, USA
[4] Pacific Ecoinformatics and Computational Ecology Lab, 1604 McGee Avenue, Berkeley, CA 94703, USA
[5] École Polytechnique, Route de Saclay, 91128 Palaiseau, France

[*] Corresponding author: anna.kuparinen@helsinki.fi; +358-40-7313120

Supplementary Methods

Lake Constance food web data set and ATN model parameterization

LC is a temperate, large (476 km^2), deep (mean depth = 101 m, max. depth 252 m), and warm-monomictic lake north of the European Alps of glacial origin with weak pelagic-benthic coupling, and little allochthonous input into the pelagic zone (Bäuerle & Gaedke 1998). The LC data set comprises long-term, high-frequency time series up to 20 years of abiotic conditions (e.g. light, temperature, mixing intensity, nutrient concentrations), species biomasses, production, and the energy and nutrient flows within the food web (Gaedke et al. 1998, 2002; de Castro & Gaedke 2008, Gaedke & Straile 1994).

To build the food web model, species were assigned to functional guilds (Table S1) sharing the same prey and predator guilds (Boit et al. 2012, Lang 1997). Plankton abundances were obtained by microscopic counting (Bäuerle & Gaedke, 1998; Gaedke 1992). Body sizes were estimated by measuring either size frequency distributions of small organisms (e.g. bacteria, heterotrophic nanoflagellates), average cell volumes of intermediate organisms (phytoplankton, ciliates, rotifers), or the individual length of large ones (crustaceans). Species size was converted to body mass in units of carbon using group-specific conversion factors (Gaedke 1992). Initial fish biomasses were based on the parameterization of Boit et al. (2012), where they were inferred from LC catch data of commercially exploited fish species (Appenzeller 1998) and sonar data (Eckmann 2010). Here, initial biomasses were split among the five age groups, but then allowed to settle into dynamics equilibriums. Robustness with respect to initial values was checked.

References

Appenzeller, A.R. Persistent large-scale heterogeneity of pelagic fish in Upper Lake Constance and its possible causes. *Arch. Hydrobiol.* 53, 303-316 (1998).

Bäuerle, E. & Gaedke, U. *Lake Constance: characterization of an ecosystem in transition* (Schweizerbartsche Verlagsbuchhandlung, 1998).

Boit, A., Martinez, N.D., Williams, R.J. & Gaedke, U. Mechanistic theory and modelling of complex food-web dynamics in Lake Constance. *Ecol. Lett.* 15, 594-602 (2012).

Brose, U., Williams, R.J. & Martinez, N. D. Allometric scaling enhances stability in complex food webs. *Ecol. Lett.* 9, 228-1236 (2006).

de Castro, F. & Gaedke, U. The metabolism of lake plankton does not support the metabolic theory of ecology. *Oikos* 117, 1218-1226 (2008).

Eckmann, R. Hydroakustische Untersuchung der Horizontal- und Vertikalverteilung der pelagischen Felchen im Bodensee-Obersee. Projektbericht zu Händen der Internationalen Bevollmächtigtenkonferenz für die Bodenseefischerei (IBKF, 2010).

Gaedke, U. The Size Distribution of Plankton Biomass in A Large Lake and Its Seasonal Variability. *Limn. Ocean.* 37, 1202-1220 (1992).

Gaedke, U., Hochstädter, S. & Straile, D. Interplay between energy limitation and nutritional deficiency: Empirical data and food web models. *Ecol. Mon.* 72, 251–270 (2012).

Gaedke, U., Ollinger, D., Bäuerle, E. & Straile, D. The impact of weather conditions on the seasonal plankton development. *Arch. Hydrobiol.* 53, 565-585 (1998).

Gaedke, U. & Straile D. Seasonal changes of trophic transfer efficiencies in a plankton food web derived from biomass size distributions and network analysis. *Ecol. Mod.* 75-76, 435–445 (1994).

Humphreys, W. F. Production and respiration in animal populations. *J Anim. Ecol.* 48, 4274-533 (1979).

Lang, M. M. *The pelagic food web of Lake Constance: An analysis based on food web theory*. Konstanzer Dissertationen 546. (Hartung-Gorre Verlag, 1997).

Nielsen, M. V. & Olsen, Y. The dependence of the assimilation efficiency in Daphnia magna on the 14C-labeling period of the food algae Scenedesmus acutus. *Limnol. Oceanogr.* 34, 1311-1315 (1989).

Skalski, G. T. & Gilliam J. F. Functional responses with predator interference: viable alternatives to the Holling Type II model. *Ecology* 82, 3083-3092 (2001).

Yodzis, P. & Innes, S. Body size and consumer-resource dynamics. *Am. Nat.* 139, 1151–1175 (1992).

Supplementary Table S1 The LC food web with size-related parameters and prey ranges. Adapted from Boit *et al.* (2012). Colors represent 8 major functional groups: phytoplankton (1-6, green), heterotrophic bacteria (7, blue), HNF (8, orange), ciliates (9-13, magenta), rotifers (14-17, dark cyan), herbivorous Crustaceans (18, light red), carnivorous crustaceans (19, dark red), fish (21-30, light blue).

ID	Name	Description	Biomass*	Body Mass†	x_i, r_i‡	FM§	Diet. ID**	Weak links
0	DOC	Pool of dissolved organic carbon	300,000	n.a.	n.a.	n.a.	n.a.	n.a.
1	Alg1	Single-cell algae, ++‡‡	5000	6.40E-05	1	a	n.a.	n.a.
2	Alg2	Large, single-cell algae or colonies, +	3000	2.56E-04	0.9	a	n.a.	n.a.
3	Alg3	Filamentous blue and green algae, --	30	3.20E-05	1.09	a	n.a.	n.a.
4	Alg4	Diatoms, algal colonies, +	5000	1.28E-04	1	a	n.a.	n.a.
5	Alg5	Small, coccal algae, ++	5000	8.00E-06	1.2	a	n.a.	n.a.
6	APP	Autotrophic picoplankton, +	20	2.50E-07	0.6	a	n.a.	n.a.
7	Bac	Heterotrophic bacteria	20,000	1.56E-08	0.04	o	0	-
8	HNF	Heterotrophic nanoflagellates, B§§	1500	8.00E-06	0.43	f/i	6-7	-
9	Cil1	Small ciliates, B	30	2.56E-04	0.14	f	6-7	-
10	Cil2	Small ciliates, B/H	150	2.05E-03	0.18	i	1,5-8	-
11	Cil3	Medium-size ciliates, H	2000	4.10E-03	0.15	f/i	1-2,5,8	2
12	Cil4	Medium-size ciliates, H	2000	8.19E-03	0.15	f	1,5,8	-
13	Cil5	Larger ciliates, O	300	6.55E-02	0.1	i	1-2,4-5,8-11	8
14	Rot1	Small rotifers, B/H	15	1.64E-02	0.13	f	1,5-8	-
15	Rot2	Medium-size rotifers, H	15	3.28E-02	0.12	f	1-9	2-4,9
16	Rot3	Large rotifers, O	50	6.55E-02	0.11	i	1-5,8-9	2-4,9
17	Asp	Large rotifers, C	50	6.55E-02	0.12	r	2-4,8-16	-
18	Cru	Mostly cladocerans (daphnids), H/O	3000	8.39E+00	0.07	f	1-16	7, 11, 14-16
19	Cyc	Cyclopoid copepods, O/C	15,000	1.05E+00	0.07	r	1-5,8- 17	14-16
20	Lep	Large, carnivorous cladocerans, C	400	6.71E+01	0.04	r	17-18	-
21	Lar1	whitefish larvae, C	500	1.28E+3	0.138	r	14-19	-
22	Lar2	perch larvae, C	500	4.56E+2	0.155	r	14-19	-
23	Juv1	whitefish juveniles, C	500	2.51E+6	0.06	r	18-20	-
24	Juv2	perch juveniles, C	500	1.35E+6	0.064	r	18-20	-
25	2yr1	2yr whitefish, C	1000	1.32E+7	0.05	r	18-20	-
26	2yr2	2yr perch, C	1000	6.42E+6	0.054	r	18-22	-
27	3yr1	3yr whitefish, C	2000	3.10E+7	0.046	r	18-20	-
28	3yr2	3yr perch, C	1000	1.37E+7	0.050	r	18-24	-
29	4yr1	4yr and older whitefish, C	2000	5.24E+7	0.043	r	18-20	-
30	4yr2	4yr and older perch, C	1000	2.14E+7	0.048	r	21-24	-

*in ($\mu gC/m^3$). †in (μgC/ind). ‡mass-specific relative growth rate *r* and metabolic rate *x* of guild *i* (1/day); scaling is done with respect to the growth rate of guild 1. §Feeding mode (a=autotroph, o=osmotroph, f=filter-feeder, i=interception feeder, r=raptorial/ambush feeder). **ID of resource guild. ‡‡Edibility (++: well-edible, +: less edible, --: edible only for specialists), §§general diet description (B=bacterivore, H=herbivore, C=carnivore, O=omnivore).

Supplementary Table S2 Summary of the ATN model parameters for Lake Constance.

Parameter	Unit	Value	Description	Reference
K	µgC/m^3	540000	Phytoplankton carrying capacity	Boit et al. 2012
x_i	1/day	0.04 - 0.18	Mass-specific metabolic rate[1]	Brose et al. 2006
r_i	1/day	0.6 - 1.09	Mass-specific growth rate for autotrophs[1]	Brose et al. 2006
c_{ij}		1	Producer competition coefficient	Boit et al. 2012
f_a		0.4	Activity metabolism coefficient	Humphreys 1979
f_m		0.1	Maintenance respiration coefficient	Humphreys 1979
y_{ij}		10	Maximum ingestion rate	Brose et al. 2006, Yodzis and Innes 1992
e_{ij}		0.66	Assimilation efficiency	Nielsen and Olsen 1989
d_{ij}	m^3/µgC	0 – 0.5	Feeding interference coefficient	Skalski and Gilliam 2001; Boit et al. 2012
q_{ij}		1.2	Functional response shape parameter	Boit et al. 2012
ω_{ij}		0 – 0.5	relative prey preference	Boit et al. 2012
p_{ij}		0 - 1	fraction of resource species shared	Boit et al. 2012
s_i		0.2	fraction of exudation	Boit et al. 2012
$B0_{ij}$	µgC/m^3	1500 - 700000	Half-saturation densities	Boit et al. 2012

[1] Relative rates with respect to guild 1; see Table S1.

Supplementary Table S3 Description of the five life stages for whitefish and perch. For details about the construction of the life stages, see methods section.

Stage	Whitefish				Perch			
	Length (cm)	Weight (g)	Metabolic rate	Diet[1]	Length (cm)	Weight (g)	Metabolic rate	Diet[1]
larvae	1.2	0.009	0.141	plankton	0.7	0.003	0.159	plankton
juveniles	13.4	19.1	0.062	plankton	9.14	10.2	0.066	plankton
2 year adult	22.7	100.0	0.051	plankton	15.1	48.7	0.056	plankton, fish larvae
3 year adults	29.8	235.2	0.047	plankton	19.3	104.4	0.051	fish larvae, juveniles
4+ year adults[2]	35.3	397.6	0.044	plankton	22.2	162.6	0.049	fish larvae, juveniles

[1] Further detail about the prey items is provided in Table S1.

[2] 4 years and older.

Supplementary Figure S1 The temporal development of age-class specific biomasses of whitefish and perch. Biomasses of 2 year old are shown with dotted line, 3 year old with thin solid line, and 4+ year old with thick solid line. Simulations without life-history changes are shown in black, while those with changing life-histories are shown in red. The beginning and the end of fishing are indicated by vertical lines.

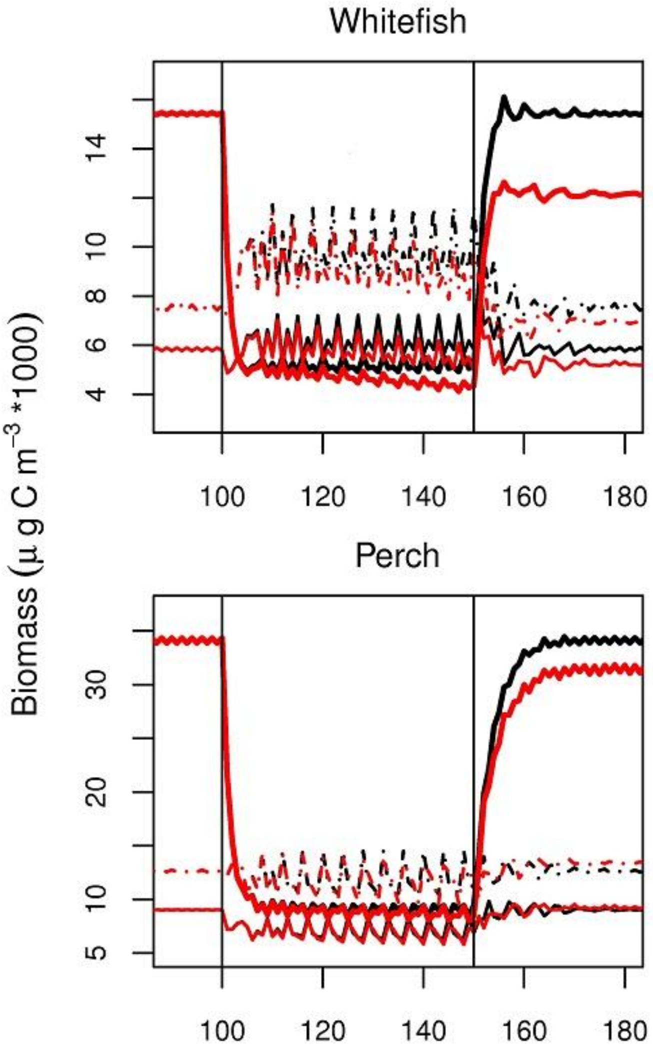

Supplementary Figure S2 Comparison of the impacts of selective and non-selective fishing with the same biomass removal. Selective fishery (black) harvests older age classes more intensively, whereas in non-selective fishery (green), fishing pressure is equal among 2, 3, and 4+ age classes. For non-selective scenario, fishing mortality rate F=0.25 ($year^{-1}$) yielded best match with the total fish biomass removal by the selective fishing scenario. The beginning and the end of fishing are indicated by vertical lines.

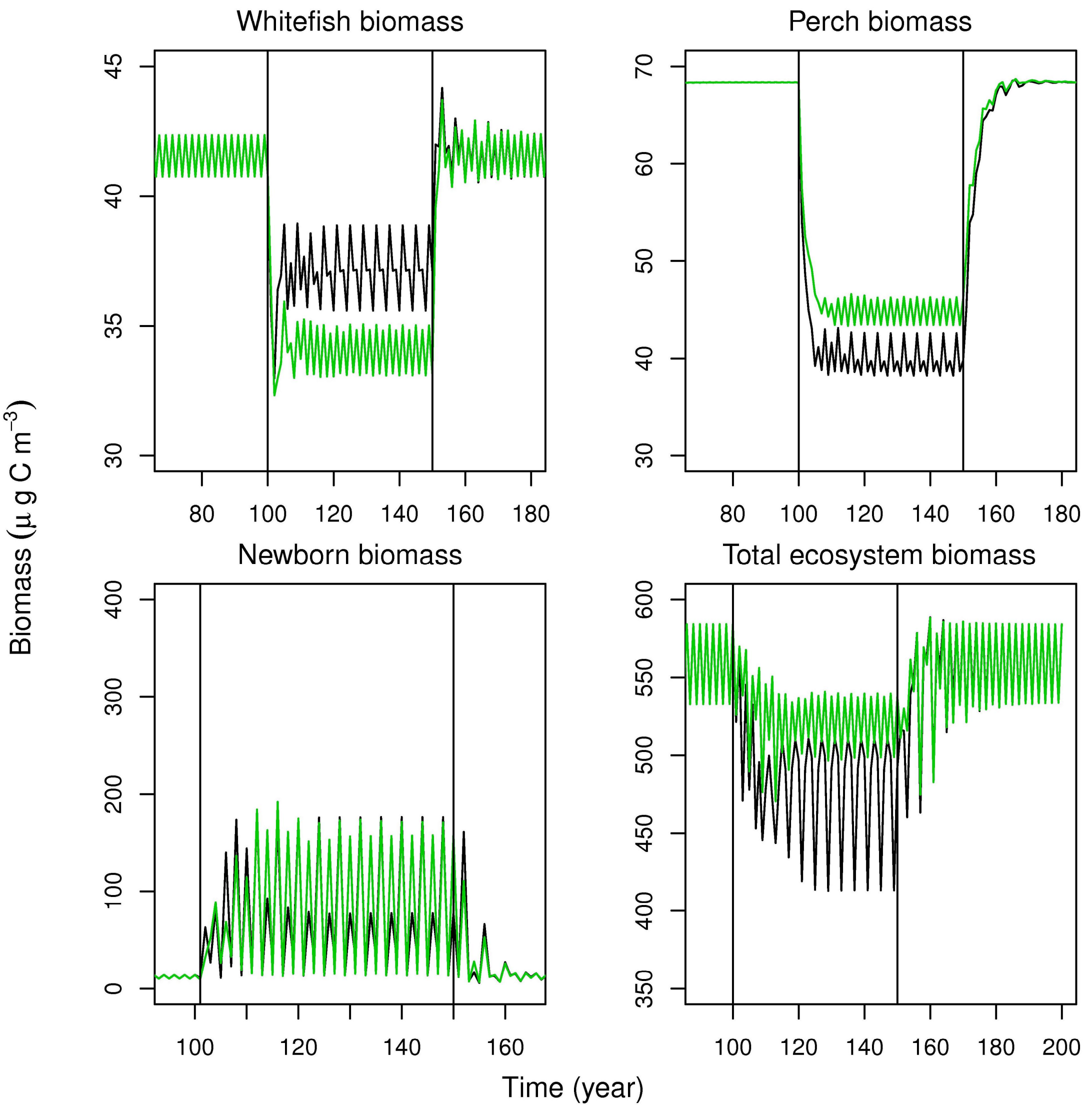

Supplementary Figure S3 Impacts of fishing selection on plankton community in the absence of life-history changes. Selective fishery scenario is shown in black and non-selective in green. Implementation of non-selective fishing is as described in Supplementary Fig. S2. The beginning and the end of fishing are indicated by vertical lines.

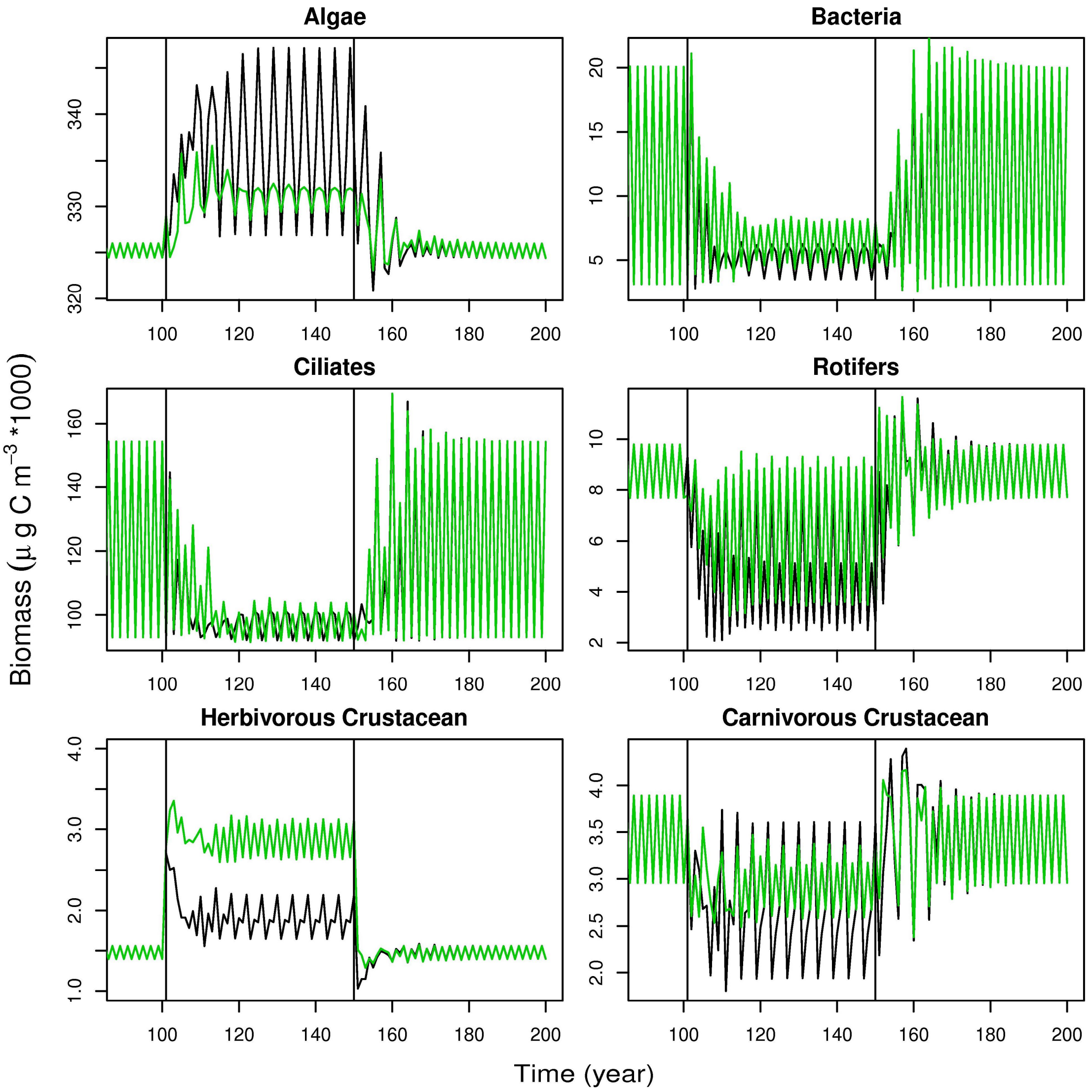

Supplementary Figure S4 Implications of life-history changes in the presences of year-to-year stochasticity in phytoplankton carrying capacity (SD: 10% of *K*). Biomasses of 2 year old fish are shown with dotted line, 3 year old with thin solid line, and 4+ year old with thick solid line. For newborn biomass and the total ecosystem biomass, smoothed patters are plotted with thick solid lines. For newborn biomass, standard deviations are similar during fishing for scenarios with (red) and without (black) life-history changes, but after the end of fishing they are about 40% higher in the presence of life-history changes, as compared to the absence of life-history changes. Similarly, variation in total ecosystem biomass is similar during fishing, but standard deviations remain about 23% higher in the presence of life-history changes after the end of fishing. Smaller amounts of stochasticity further emphasized the role of life-history changes, whereas if SD of the stochasticity about *K* was set to 15% of *K*, increased variability associated to fish life-history changes was largely masked by the variability driven by *K*.

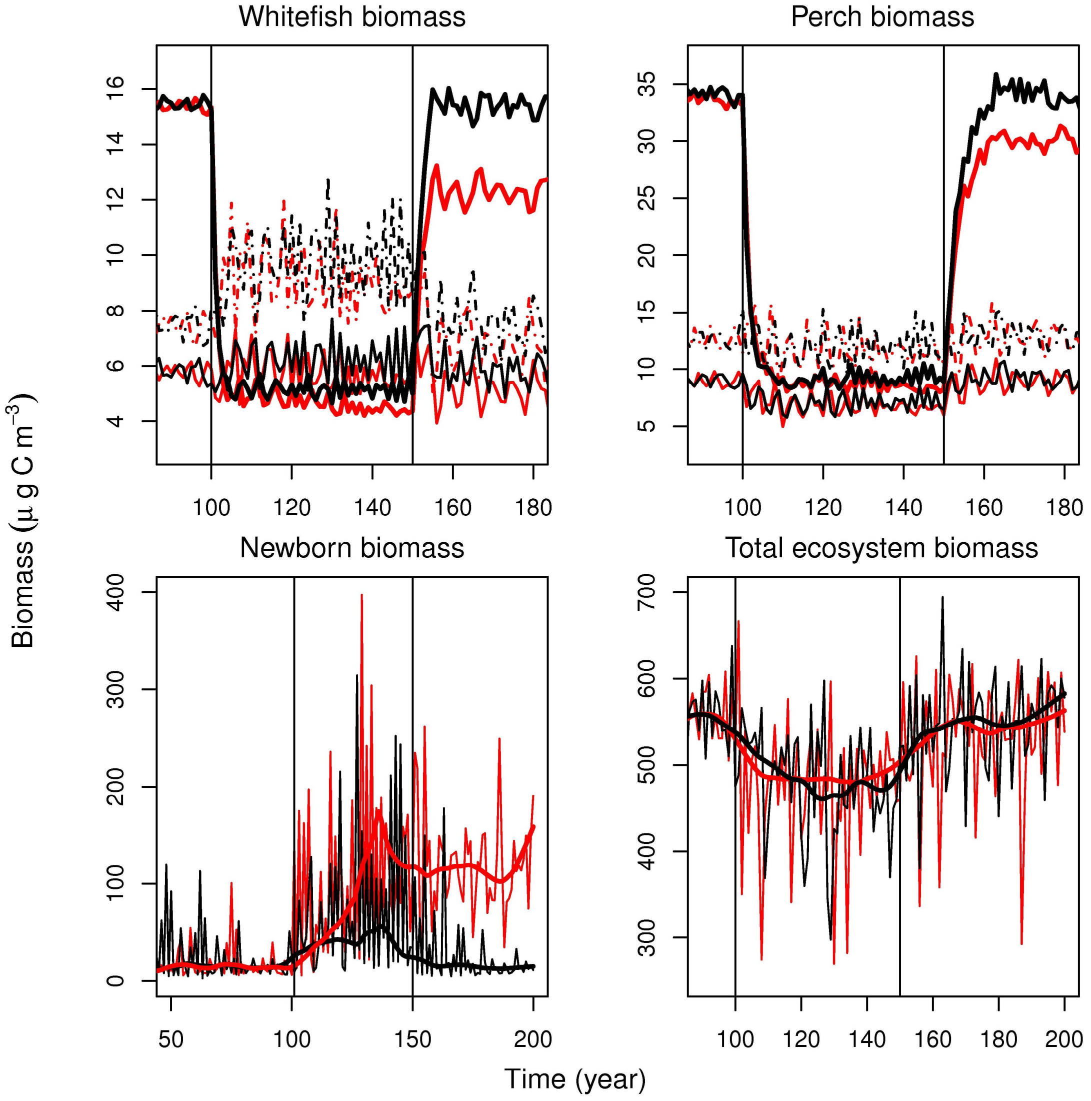

Supplementary Figure S5 Biomasses of six major plankton groups in the presence of year-to-year stochasticity with (red) and without (black) life-history changes. Stochasticity was implemented similarly as described in Supplementary Fig S4. Smoothed patterns are plotted with solid thick lines.

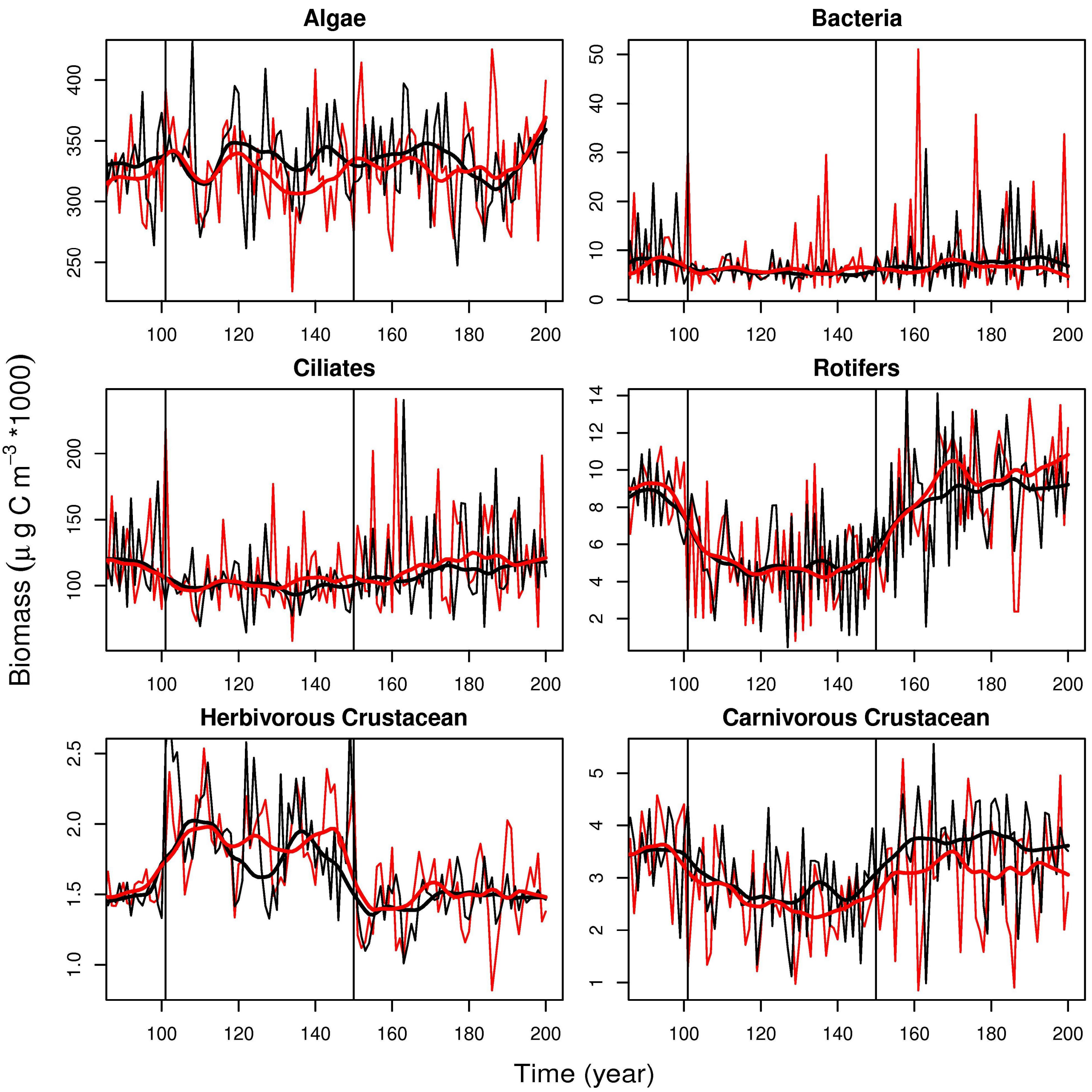

Supplementary Figure S6 The role of fish life-history changes in the absence of biomass removal by fishing. The scenario with life-history changes (reducing fish body size and the age at maturation) is shown in red and the one without life-history changes in black. Biomasses of 2 year old fish are shown with dotted line, 3 year old with thin solid line, and 4+ year old with thick solid line.

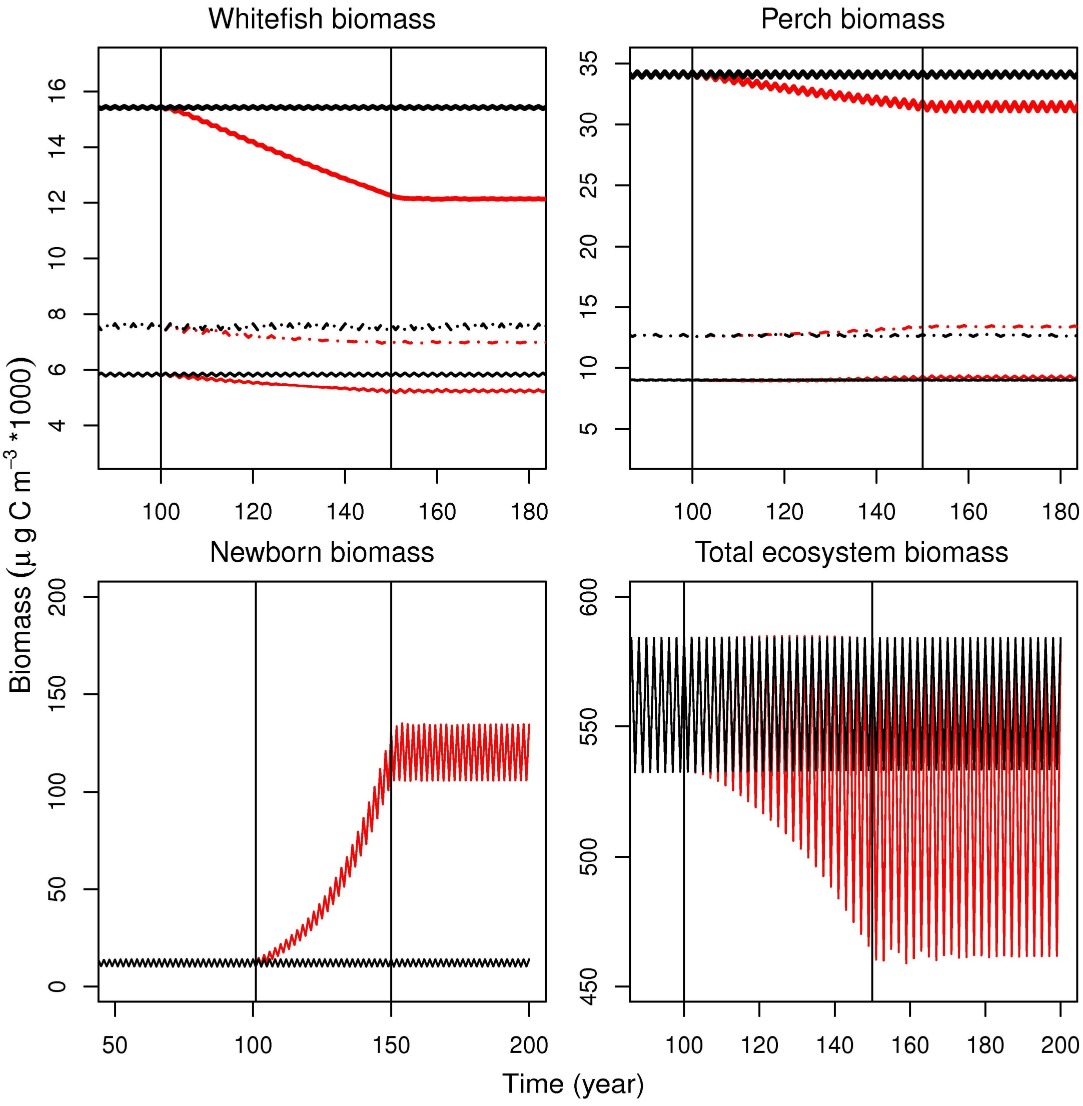

Supplementary Figure S7 The impacts of fish life-history changes on six major plankton groups in the absence of biomass removal by fishing. The scenario with life-history changes is shown in red and the one without life-history changes in black.

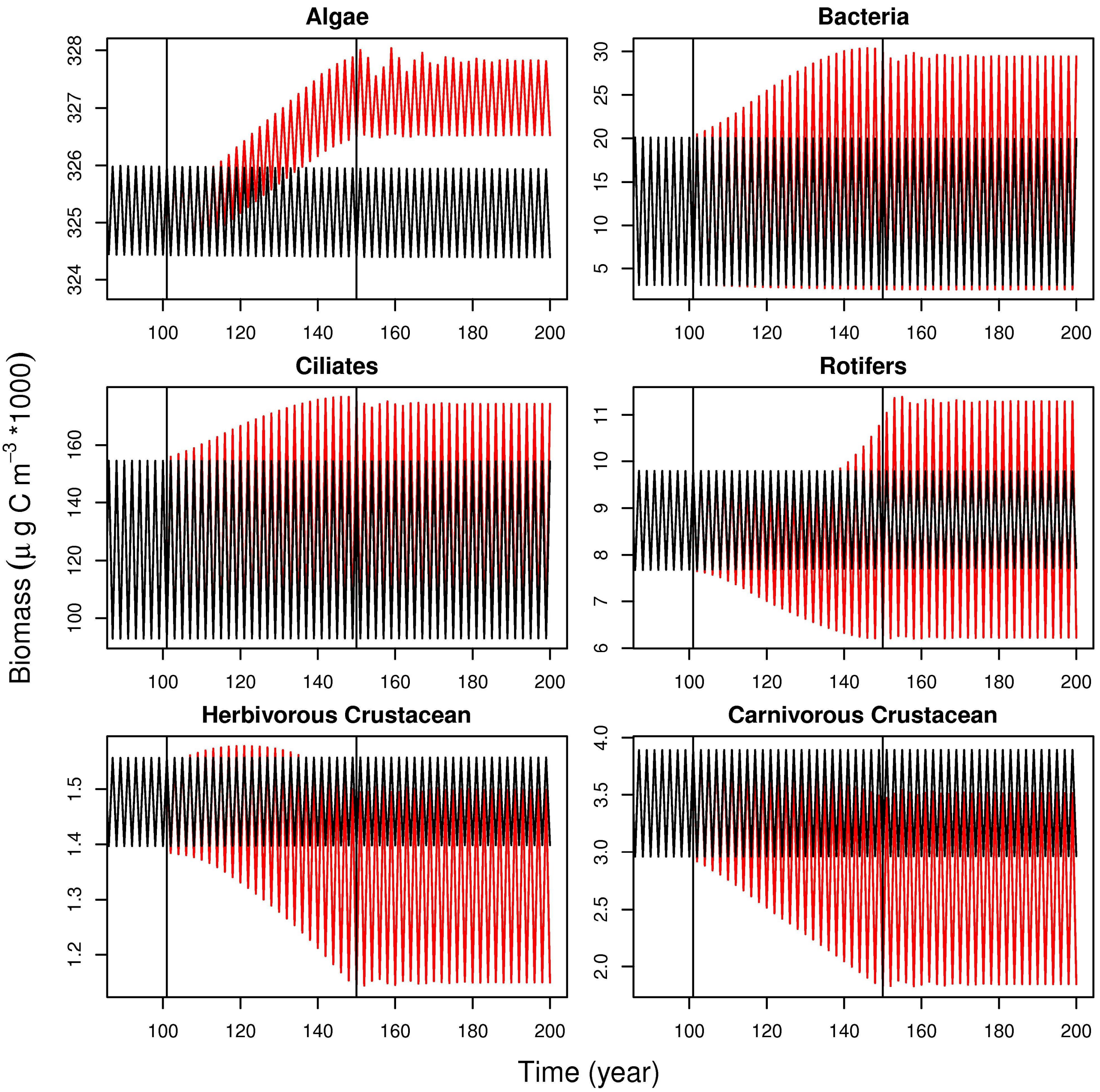

Supplement: Supplementary Information [file srep22245-s1.pdf]
